# A New Anthelmintic

**Published:** 1880-04

**Authors:** 


### A NEW ANTHELMINTIC.

The Ocinum Baselicum, a plant known in Buenos Ayres under the name of Albochaca, has an action of such a nature that the worms in every stage of development rapidly leave their location after the juice reaches them. Its use is so much more to be recommended, since in the event no worms are present, no injurious effect results from the plant, but a laxative and disinfectant action as the only result. Fifty grammes of the juice is given, followed in two hours by a dose of castor oil. A free discharge of the worms may be expected.

The above observations of Dr. Lemor, and the results obtained are very encouraging, and invite further investigation, the more since the number of anthelmintics is limited, and their action often unsatisfactory.—*Med. Neuigk.*

